# Origin, genetic structure and evolutionary potential of the natural hybrid *Ranunculus circinatus* × *R. fluitans*

**DOI:** 10.1038/s41598-023-36253-7

**Published:** 2023-06-03

**Authors:** J. Zalewska-Gałosz, M. Kwiatkowska, J. Prančl, K. Skubała, M. Lučanová, D. Gebler, K. Szoszkiewicz

**Affiliations:** 1 Institute of Botany, Faculty of Biology, Jagiellonian University, Gronostajowa 3, 30-387 Kraków, Poland; 2 Institute of Botany, Czech Academy of Sciences, Zámek 1, 252 43 Průhonice, Czech Republic; 3 Department of Ecology and Environmental Protection, Poznan University of Life Sciences, Wojska Polskiego 28, 60-637 Poznań, Poland; 4 Department of Botany, Faculty of Science, University of South Bohemia, Branišovská 31, 370 05 České Budějovice, Czech Republic

**Keywords:** Plant evolution, Plant hybridization, Polyploidy in plants

## Abstract

Understanding the genetic variability of hybrids provides information on their current and future evolutionary role. In this paper, we focus on the interspecific hybrid *Ranunculus circinatus* × *R. fluitans* that forms spontaneously within the group *Ranuculus* L. sect. *Batrachium* DC. (Ranunculaceae Juss.). Genome-wide DNA fingerprinting using amplified fragment length polymorphisms (AFLP) was employed to determine the genetic variation among 36 riverine populations of the hybrid and their parental species. The results demonstrate a strong genetic structure of *R. circinatus* × *R. fluitans* within Poland (Central Europe), which is attributed to independent hybridization events, sterility of hybrid individuals, vegetative propagation, and isolation through geographical distance within populations. The hybrid *R. circinatus* × *R. fluitans* is a sterile triploid, but, as we have shown in this study, it may participate in subsequent hybridization events, resulting in a ploidy change that can lead to spontaneous fertility recovery. The ability to produce unreduced female gametes of the hybrid *R. circinatus* × *R. fluitans* and the parental species *R. fluitans* is an important evolutionary mechanism in *Ranunculus* sect. *Batrachium* that could give rise to new taxa.

## Introduction

Interspecific hybridization plays an important role in the evolution of many plant lineages^1–3^. It can lead to a variety of outcomes affecting diversity, through speciation^4–6^, gene flow from one taxon to another (introgression)^7–9^, fusion of previously divergent taxa^10^, or extinction of one or both parental taxa^11^. In this context, understanding the genetic variability of hybrids could provide information on both their current and future evolutionary role.

A plant group where hybridization significantly shapes taxonomy is *Ranunculus* L. sect. *Batrachium* DC. (Ranunculaceae Juss.), hereafter referred to as *Batrachium*^12^. The section comprises ca. 30 species of aquatic plants occurring worldwide^13^. Some of the species are stable, fertile allopolyploids (e.g., *R. aquatilis* or *R. schmalhausenii*), however with still recognizable signs of hybridization confirmed by molecular markers^14–16^. The actual number of hybrid taxa within the group is hard to estimate due to unclear morphological boundaries between species and hybrids, hybrid cytodemes with unknown taxonomic status, and insufficient knowledge of *Batrachium* diversity in some areas of the world^13^. In general, *Batrachium* hybrids are defined by sterility or reduced fertility and intermediate morphology. Frequently, they are characterized by a genome size that is intermediate between putative parental taxa^13,16^. The lower fitness of the hybrids, manifested mainly by sterility or reduced fertility, is compensated by vegetative reproduction commonly achieved by aquatic plants^17^. Macrophytes can form large numbers of vegetative propagules in a short time. Moreover, compared to seeds, i.e. turions or other vegetative fragments are able to photosynthesize and take up nutrients, which facilitates successful population expansion^18^. It is well-known that some sterile hybrids of macrophytes can persist at their sites for hundreds of years spreading only vegetatively^19–21^. The vast majority of these localities are known for northern Europe, where, due to the climatic conditions, the sustainability of the aquatic ecosystems is ensured. In astatic ponds or periodic rivers of lower latitude climates taxa unable to produce seeds cannot survive long periods of water shortage. Studying the origin, genetic variation and evolutionary potential of hybrids can help to understand the consequences of ongoing changes in the distribution of global biodiversity^22^.

One of the *Batrachium* hybrids, namely *Ranunculus circinatus* × *R. fluitans*, has received special attention from taxonomists^16,23,24^. This taxon was first reported from Germany^25^, however, due to the lack of easily detectable diagnostic features, it was not later included in subsequent taxonomic studies^26^. Recently, the hybrid was confirmed based on molecular evidence from Bavaria^16^, several sites in Poland^23^, and Lithuania, from where it was formally described as *R.* × *redundans* AA. Bobrov, Butkuvienė et Sinkevičienė^24^. It is possible that this hybrid is much more common in Europe than it appears from the published data, but it is overlooked due to its significant morphological similarity to other hybrids of *R. fluitans,* and the lack of common investigation of molecular features, which facilitates identification of morphologically ambiguous taxa.

**Figure 1:**
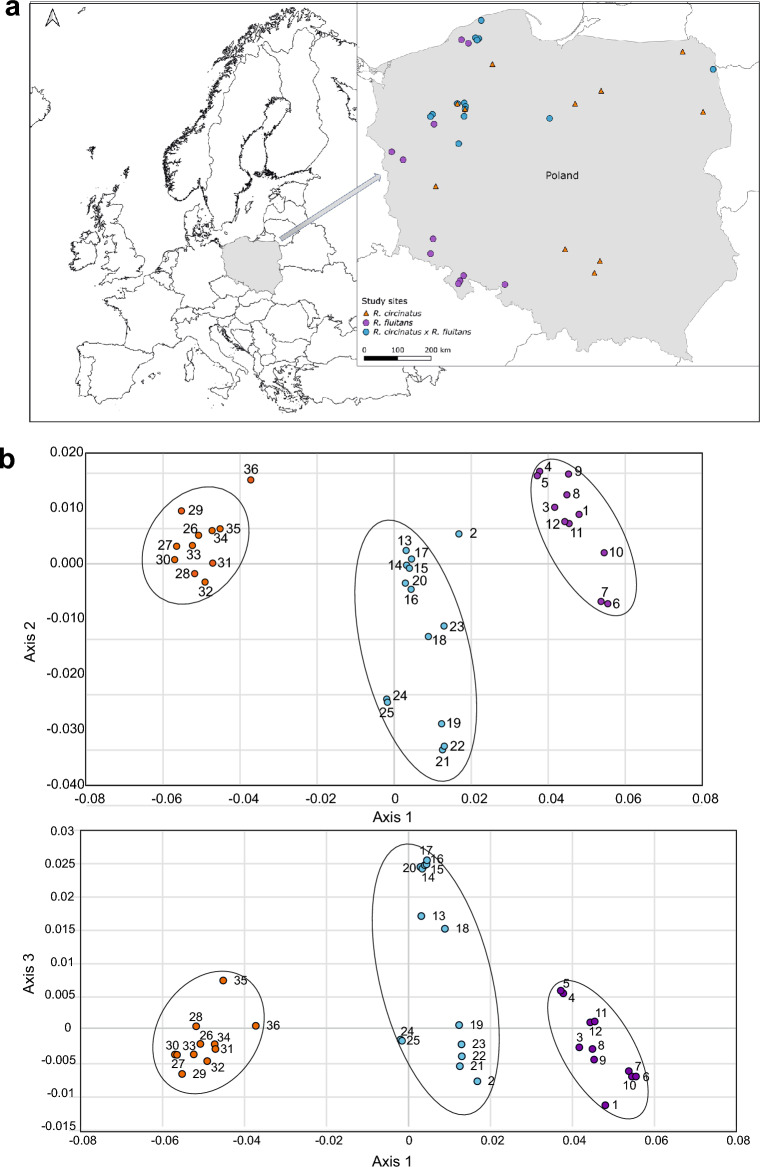
(**a**) map of the sampled populations of the hybrid *Ranunculus circinatus* × *R. fluitans* (green circles) and its parental species: *R. circinatus* (orange triangles) and *R. fluitans* (violet circles). The figure was generated using QGIS Software ver. 2.30.2-Odense https://www.qgis.org/en/site/, the map of Europe was taken from Wikimedia Commons https://commons.wikimedia.org/wiki/File:Borders_Europe_Map_HD.png (**b**) Principal Coordinates Analysis (PCoA) using unbiased Nei’s genetic distances of the amplified fragment length polymorphism (AFLP) dataset. Colours of the points denote the populations of taxa: orange—*R. circinatus*, violet—*R. fluitans*, blue—the hybrid *R. circinatus* × *R. fluitans*. Populations numbers are explained in Supplementary Table S1.

In Poland (Central Europe) the hybrid *R. circinatus* × *R. fluitans* was identified in twelve rivers flowing in different northern parts of the country and belonging to seven different catchments (Fig. 1a). In a previous study, it was shown that the ecological properties of *R. circinatus* × *R. fluitans* are different from the parental species, but more similar to *R. fluitans* than *R. circinatus.* Additionally, it was shown that the hybrid prefers rhithral conditions and large watercourses. The hybrid is typically found in lake outflows or other ecotones, where riverine *R. fluitans* and standing water *R. circinatus* co-occur. The hybrid can also grow in rivers where fast-flowing and standing water sections are found within a short distance. Based on the direct sequencing of ITS and cpDNA regions *rpl32-trnL* and *psbE-petL*, the parentage of this hybrid was confirmed and *R. fluitans* was proved to be a donor of plastid DNA^23^. The same direction of crossing was confirmed by Bobrov et al.^24^ for the hybrid individuals occurring in the Lithuanian rivers.

The intermediate morphological characteristics, such as the variable shape of the nectar pits, and the widespread sterility of the hybrid individuals suggest that its populations represent the F1 generations. However, it is not yet proven and remains a hypothesis with a model of *R. circinatus* × *R. fluitans* formations (i.e., unique versus multiple hybridizations). The number of independent origins is still unknown.

The primary aim of our study was to explore whether populations of *R. circinatus* × *R. fluitans* occurring in Poland have a common mode of origin, or, if not, what their genetic structure looks like. Moreover, the specific aims are focused on: (1) examining hybrid fertility (development of gametophytes, pollen viability), (2) assessing the ploidy level, and (3) testing if the observed morphological variation reflects backcrossing with the parental species.

The implementation of the described goals will allow us to draw conclusions about the frequency of this hybrid formation in nature, the methods of its propagation, and the possible impact of this hybrid on the taxonomic diversity within *Batrachium*.

## Results

### Genetic structure and variation of *R. circinatus* × *R. fluitans* in natural populations

Principal Coordinates Analysis (PCoA) based on unbiased Nei's genetic distances and the whole data set showed three genetic groups reflecting a priori determined taxa (Fig. 1b). The first three axes explained 85.89% of the variation (71.31%, 8.7%, 5.88% for the first, second and third, respectively). The genetic groups were internally consistent except for one outlier in *R. circinatus* group, namely population 36 (GID), and one in the *R. circinatus* × *R. fluitans* group, population 2 (SZL). A group of populations comprising the hybrid *R. circinatus* × *R. fluitans* individuals is placed in an intermediate position in the genetic space, and between the parental species groups. The hybrid group was the most genetically diverse.

Analysis of the genetic variation of the hybrid *R. circinatus* × *R. fluitans* based on AMOVA indicated that most of the variation is attributed to among-populations, i.e., 96%, relative to 4% for within-population variation (PhiPT = 0.936; p = 0.001, Table 1).

**Table 1:** Analysis of molecular variance (AMOVA) based on AFLP markers for 13 populations of the hybrid *R. circinatus* × *R. fluitans.*

Variation	df	SS	MS	Est. var	Per. var	PhiPT	P
Among populations	12	3032.908	252.742	48.739	96%		
Within populations	54	102.167	1.892	1.892	4%		
Total	66	3135.075		50.631	100%	0.963	0.001

*df* degrees of freedom, *SS* sum of squares, *MS* mean square, *Est. var.* estimated variance, *Per. var.* a percentage of variation.

**Figure 2:**
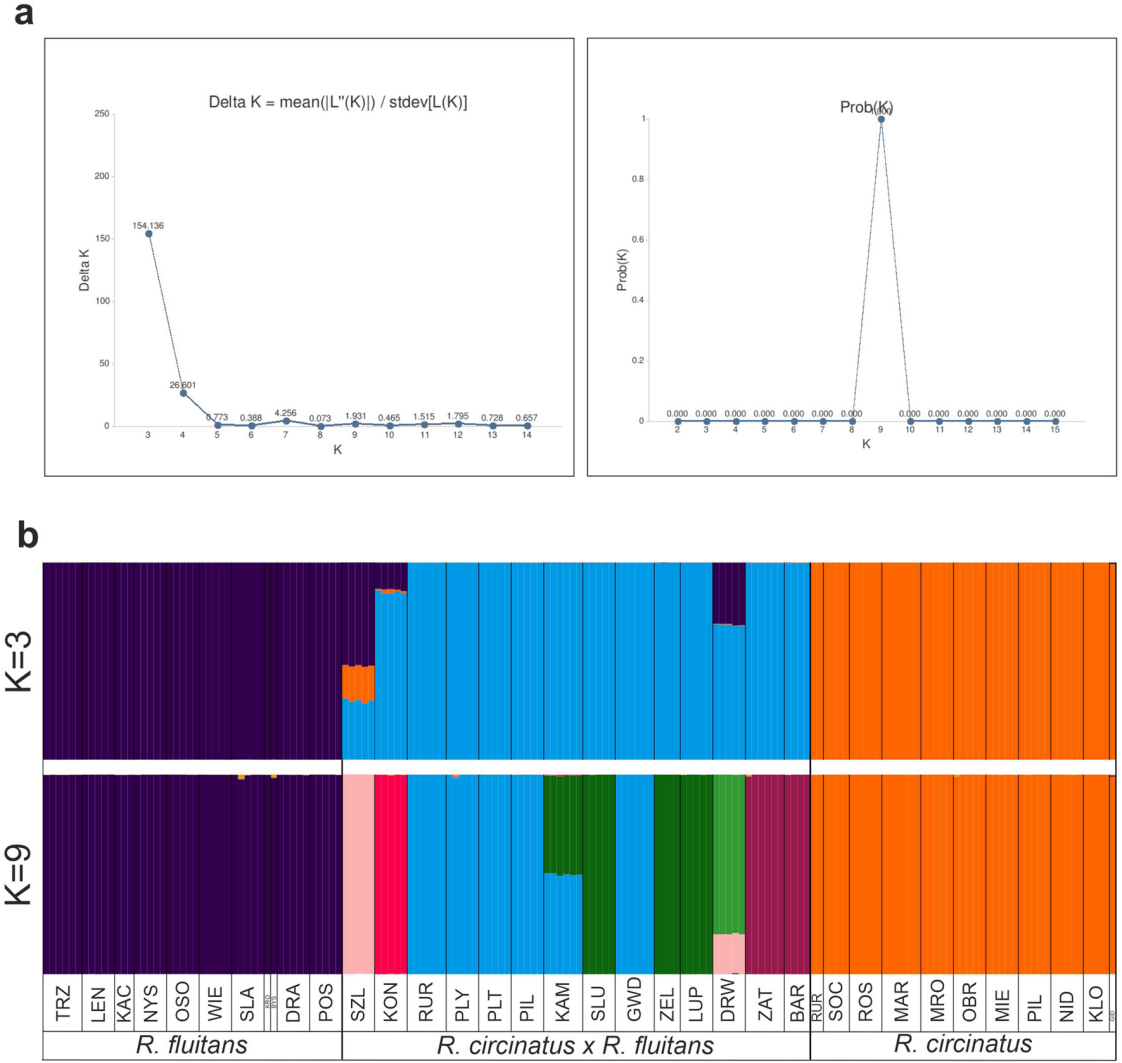
Bayesian analysis (STRUCTURE); (**a**) values of K obtained based on delta*K* and mean likelihood Ln (K); (**b**) bar graphs of individuals for K = 3 and K = 9, correlated allele frequencies and admixture. Colours denote the probable ancestry coefficient of the individual and each genetic cluster. Populations are separated by vertical lines, their codes are explained in Supplementary Table S1. Studied taxa are indicated below population codes.

In the STRUCTURE analysis of AFLP data, the optimal number of populations groups (*K*) was three according to Evanno et al.^27^, but the highest log-likelihood value^28^ was found for *K* = 9 (Fig. 2a). The first division (*K* = 2) was formed between populations of *R. circinatus* and *R. fluitans* together with *R. circinatus* × *R. fluitans* (Supplementary Fig. S1). For *K* = 3, three groups were formed reflecting the analyzed taxa, namely *R. fluitans, R. circinatus,* and the hybrid *R. circinatus* × *R. fluitans.* Within the hybrid *R. circinatus* × *R. fluitans* genetic group, three populations showed a significant admixture of *R. fluitans* markers: there were populations: 2 (SZL), 23 (DRW), and 13 (KON). For *K* > 4 < 9 the genetic groups of *R. fluitans* and *R. circinatus* were still homogeneous and fully corresponded with the species affiliations, while the hybrid group became more diversified (Supplementary Fig. S1). For *K* = 9, populations of the hybrid are split into seven groups: the biggest one was formed by five populations in the Gwda River basin: 14 (RUR), 15 (PLY), 16 (PLT), 17 (PIL), and 20 (GWD). The second group was formed by three populations: 19 (SLU), 21 (ZEL), and 22 (LUP). The third group included two populations from the Drawa River: 24 (ZAT) and 25 (BAR). The last four groups comprised single populations: 2 (SZL), 13 (KON), 23 (DRW), and 18 (KAM). Individuals from population 18 (KAM) showed a high degree of admixture of two hybrid gene pools (Fig. 2b).

**Figure 3:**
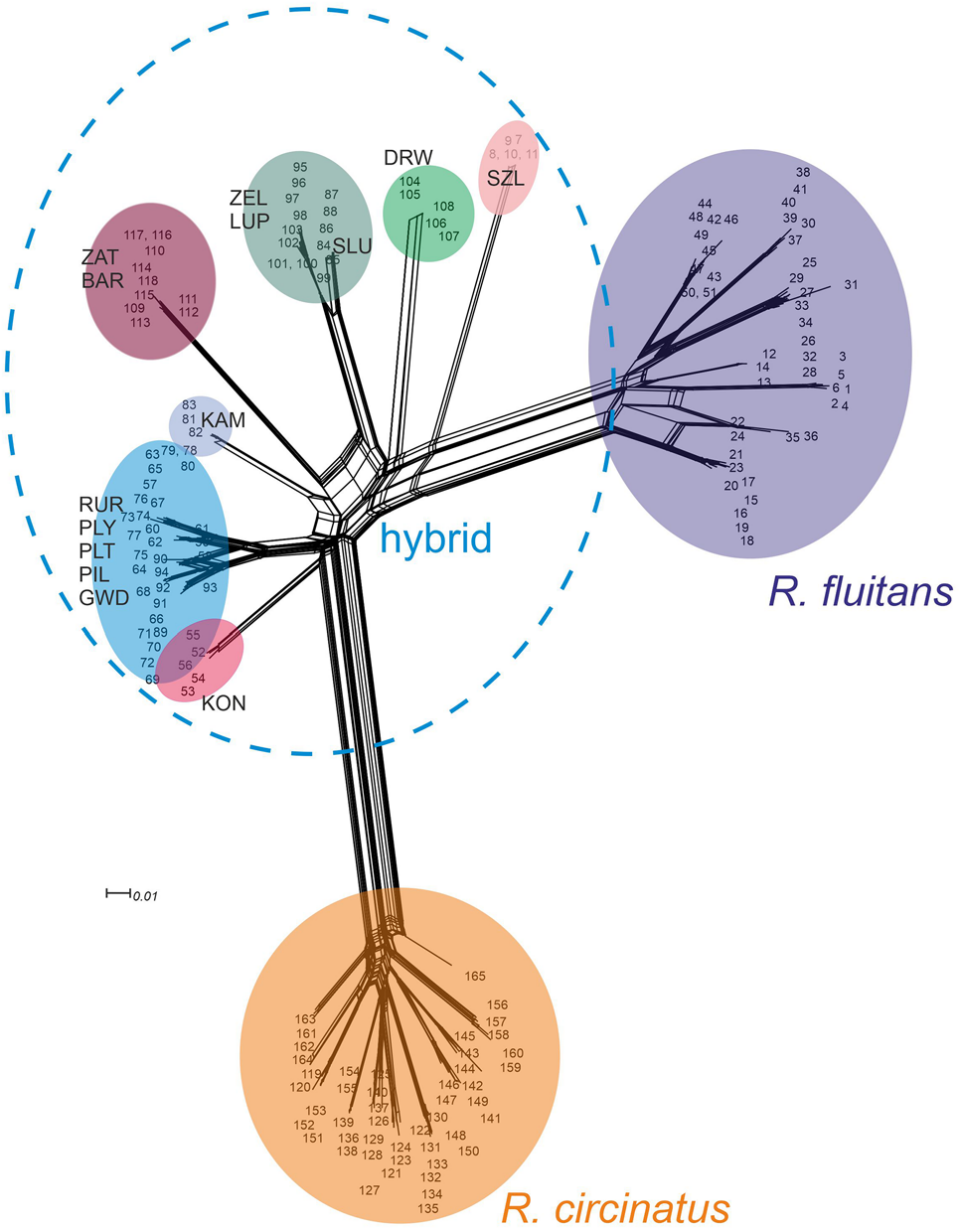
NeighbourNet analysis of the amplified fragment length polymorphism (AFLP) dataset of *R. circinatus, R. fluitans*, and its hybrid *R. circinatus* × *R. fluitans*. Shaded polygons indicate genetic groups, dashed line underlines the heterogenic cluster of *R. circinatus* × *R. fluitans* discussed in the text. Bootstrap support for all main splits is > 90. Codes of *R. circinatus* × *R. fluitans* populations are explained in Supplementary Table S1.

NeighbourNet analysis revealed two coherent genetic groups that reflected taxonomic affiliations of individuals to the species *R. circinatus* and *R. fluitans*, respectively, and a third heterogeneous group placed between and consisting of hybrid individuals *R. circinatus* × *R. fluitans* (Fig. 3). Within this group, seven splits were distinguished. The most separated position, and at the same time the closest to *R. fluitans* group encompasses population 2 (SZL). The rest of the hybrid populations are placed just in the middle, between parental clusters, which demonstrates that these populations comprised F1 generations of *R. circinatus* × *R. fluitans*. It is clear, however, that each cluster is genetically distinct, and originated from a separate hybridization event. All these internal splits are compatible with the STRUCTURE analysis in which heterogeneity of the genetic group of *R. circinatus* × *R. fluitans* is also revealed.

### Analysis of the correlation between genetic and geographic distance

Fourteen populations of the hybrid *Ranunculus circinatus* × *R. fluitans* grew in 12 rivers flowing through different parts of northern Poland and were assigned to seven independent genetic clusters that stand out in separate catchment areas (Fig. 3). To understand the effects of geographic distance on population, we applied the Mantel test, which indicated a significant, positive correlation between genetic differentiation and geographic distance for the total data set (R = 0.27, p = 0.01; Supplementary Fig. S2), as well as for *R. circinatus* × *R. fluitans* populations (R = 0.35, p = 0.01, Supplementary Fig. S3). These results suggest that geographic distance is one of the factors affecting the existing pattern of genetic distribution. This result is in line with the STRUCTURE and NeighbourNet analyses outputs, revealing that all genetic clusters within the *R. circinatus* × *R. fluitans* group were created by independent hybridization events.

However, two populations in genetic analyses were placed in groups not correlated with geographic distance. Individuals from the LUP population have the same genetic characteristics as the populations from the ZEL (Fig. 3), despite the sites of these populations occurring in rivers belonging to separate catchments (the Łupawa and the Słupia catchment, respectively). These populations are separated by about 40 km in a straight line. The shoot fragments of the hybrid are likely to have been transferred by long-distance transport between these population, either by birds, or nonintentional or intentional human activities (i.e. reintroduction, see “Discussion”).

### Chromosome number

**Figure 4:**
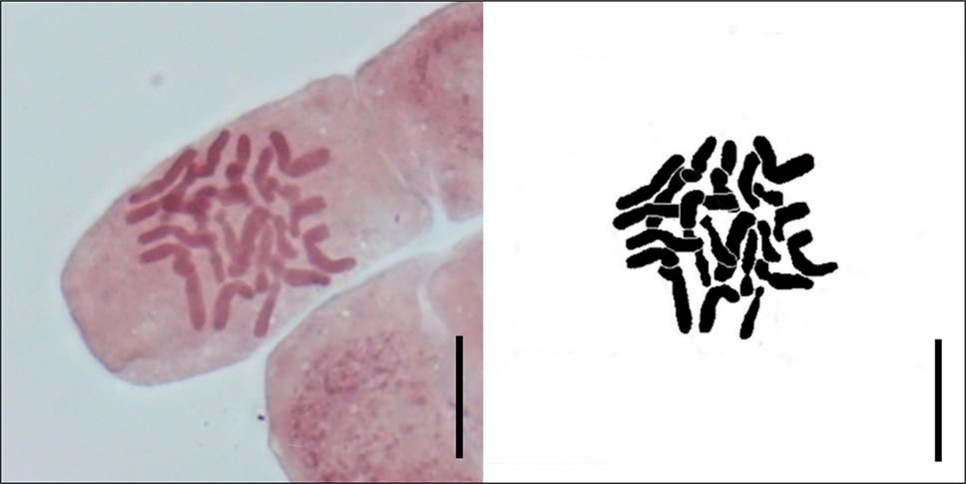
Mitotic metaphase chromosomes (photograph and its interpretation) of *Ranunculus circinatus* × *R. fluitans* from RUR population. 2n = 24. Scale bar = 10 µm.

In the roots of all tested plants, cells at the interphase stage prevailed in the apical root meristem. Prophase was the most frequent phase of cell division. In the late prophase and in the metaphase the chromosome was counted, 2n = 24 (Fig. 4), corresponding to triploid ploidy level (x = 8). Several of the metaphase plates had chromosome patterns that could not be counted. Anaphases and telophases were observed very rarely.

### Genome size

Genome size (GS) data are summarized in Table 2. Of the three taxa that were studied, only *R. circinatus* shows a uniform genome size. In contrast, *R. fluitans* possesses two discrete cytotypes, corresponding to diploids and autotriploids, as can be inferred from the comparison of both mean 2C-values (ratio 1.48). While diploid *R. fluitans* turned out to be widespread across the populations, only a single population (KAC) was found to be triploid. The morphologically strange population TRZ, forming intermediate leaves with flattened leaf segments, shows a GS identical to diploid *R. fluitans*. This result is in line with the other analyses based on AFLP data: PCoA (Fig. 1b), STRUCTURE (Fig. 2b), and NeighbourNet (Fig. 3). Of the samples assigned to the hybrid of two diploid species *R. circinatus* × *R. fluitans*, surprisingly none seems to be diploid. The GS of most hybrid populations corresponds well to the triploid cytotype consisting of two chromosome sets from *R. fluitans* and one from *R. circinatus* (calculated vs. observed GS 6.71 vs. 6.69 pg). A single population of the presumed hybrid (SZL) shows the largest GS within our dataset. This cytotype could possibly belong to an allotetraploid taxon informally referred to as “*R. penicillatus* B” in previous studies^16,29^, consisting of three chromosome sets from *R. fluitans* and one from *R. circinatus* (calculated vs. observed GS 8.63 vs. 8.77 pg, i.e., 1.3% difference). The observed genome sizes for individual taxa correspond well to the values given for the same taxa by Koutecký et al.^16^. The only exception is the population SZL, as its 2C-value does not quite match the GS published for “*R. penicillatus* B” by Koutecký et al.^16^, namely 5.8% difference.

**Table 2:** Summary of flow cytometric genome size estimations. N: number of individuals, only individuals measured repeatedly on different days are included. Ratio: mean ratio with the internal standard *Bellis perennis*. 2C: mean genome size in pg of DNA. SD: standard deviation. Min, Max: minimum and maximum 2C values. Ploidy: expected ploidy level. 2C^16^: mean genome size for the respective taxon estimated by Koutecký et al.^16^; in that paper, ploidy level of all five taxa was confirmed by chromosome counting.

Taxon/cytotype	N	Ratio	2C	SD	Min	Max	Ploidy	2C^16^
*R. fluitans* (2×)	31	1.14	3.84	0.06	3.72	3.95	2x	3.84
*R. fluitans* (3×)	3	1.69	5.72	0.02	5.71	5.75	3x	5.75
*R. circinatus*	18	1.70	5.74	0.04	5.68	5.81	2x	5.67
*R. circinatus* × *R. fluitans*	32	1.98	6.69	0.09	6.46	6.83	3x	6.65
*R. circinatus* × *R. fluitans* (SZL)	3	2.59	8.77	0.02	8.75	8.79	4x	8.29

### Sexual reproduction of *R. circinatus* × *R. fluitans*

#### Pollen viability/stainability

Pollen grains were observed in 20 flowers, namely: 15 from KON (8,298 pollen grains), one from RUR (113 pollen grains), and four from SZL (4192 pollen grains).

In flowers from KON pollen grains were degenerated in 99.6 ± 0.35% (15 flowers/8298 pollen grains). Stained cells rarely appeared on the slide, and pollen viability was extremely low, reaching only 0.36%. The diameter of stainable pollen grains was 24.98 ± 4.72 µm. Most frequently, empty or only slightly stained pollen grains were observed and were of variable size. In open flowers, most often 1-nucleate grains/microspores or microspores enclosed in tetrads, occasionally in triads, pentads were also observed. All these structures were inhibited in development. In other anthers, grains were grouped together and were not countable.

Of the four flowers tested from RUR, pollen was countable in only one flower, but even then only a few pollen grains were found (113), and all were 100% degenerated. There were no countable pollen grains in the remaining three flowers, only groups of clumped cells stuck to the anther wall were observed.

From three flowers at the *preanthesis* stage (PIL, RUR, SLU) there were no properly developed microspore tetrads. Only incorrectly formed stamens were observed, either empty anthers or sticky material indistinguishable from the anther walls. It was not possible to observe the course of meiosis.

**Figure 5:**
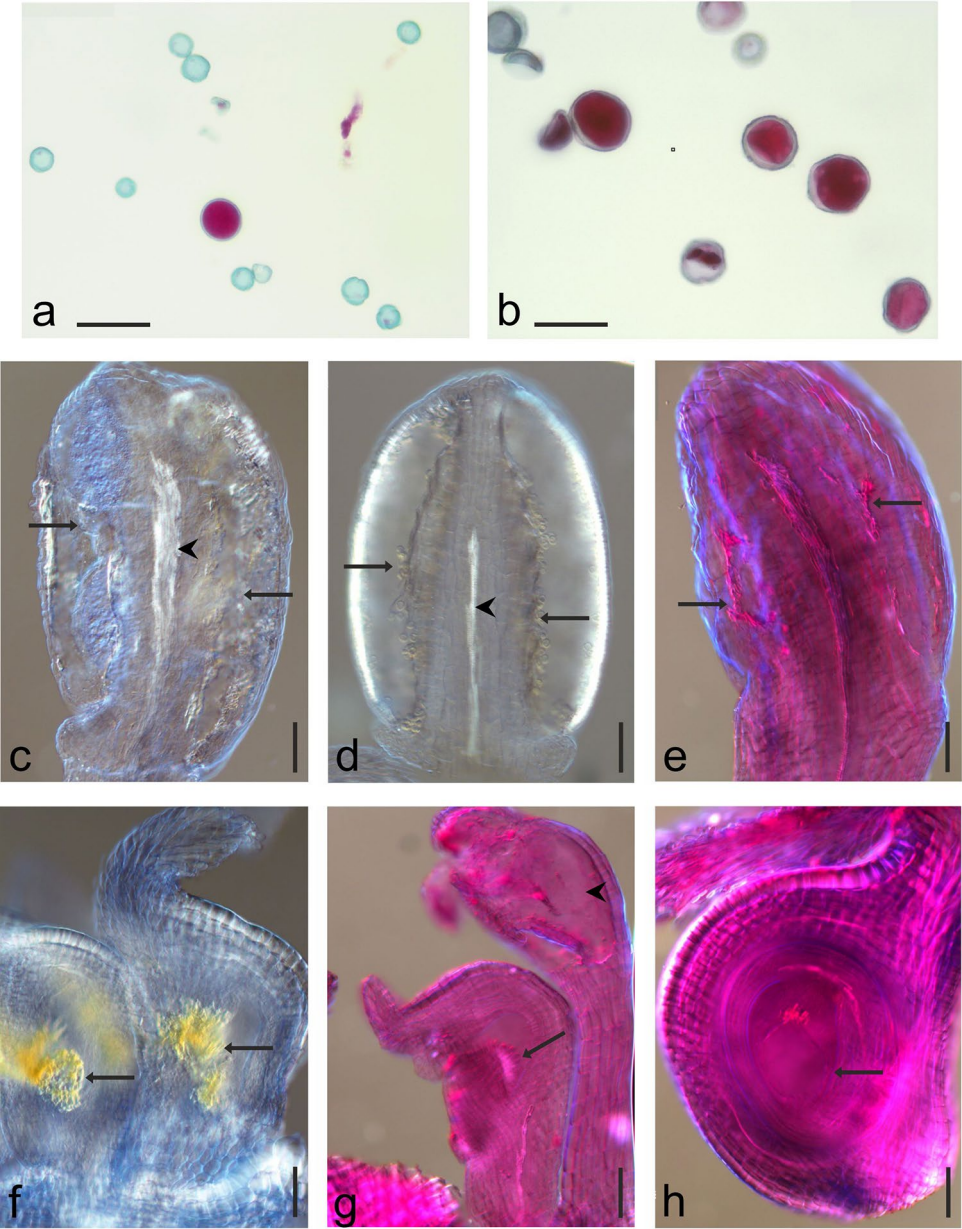
Pollen grains stainability and generative structures development in *Ranunculus* hybrid; (**a**,**b**) stainability/viability of pollen grains after staining with Alexander dye. Red stained viable pollen grains, green stained non-viable pollen grains; (**a**) site KON, (**b**) site SZL. Anthers structure after cleared with methyl salicylate: (**c**) anther from the flower at *preanthesis* stage, pollen sacs filled with undifferentiated, glued tissue (arrows), vascular strand visible in the center, between pollen sacs (arrowhead), site RUR; (**d**) anther from the flower at *anthesis* stage, pollen sacs with empty pollen close to the wall (arrows), empty rest of the chamber, anther wall properly developed with fibrous endothelium, vascular strand visible in the center, between pollen sacs (arrowhead), site KON; (**e**) anther in the stage of degeneration from the flower at *postanthesis* stage, pollen sacs with degenerated generative tissues (arrows), site PIL. Pistil structure from RUR site, after cleared with methyl salicylate: (**f**) pistils from the flower at *preanthesis* stage, properly developed pistils with degenerated ovule (arrows); (**g**) pistils from the flower at *postanthesis* stage, properly developed pistil with no ovule, instead of an ovule, finger-like elongated cells grew on the placenta (arrow), above the pistil, the stamen is abnormally developed with empty pollen sac (arrowhead); (**h**) pistil from the flower at *postanthesis* stage, properly developed pistil with an ovule, embryo sac visible without any cells (arrow). Scale bars: (**a**,**b**) 50 µm; (**c**–**h**) 100 µm.

In most likely tetraploid individuals from SZL, pollen grains were more viable than those from KON and RUR. From 12 flowers only in four pollen grains were observed. The stainability/viability of pollen grains was 17.18% ± 3.17, and the diameter of pollen grains reached 33.35 ± 3.80 µm (four flowers/4192 pollen grains). In the other eight flowers, pollen grains were mostly shrunken, glued together, and stuck to the anther walls and uncountable. In single anthers, there were several non-stained and stained grains, as in other studied populations (Fig. 5a,b; Supplementary Fig. S4a–c).

### General embryological features of *Ranunculus*

In the genus *Ranunculus* one anatropous, unitegmic, a pseudocrassinucellar ovule occurs in the ovary. The archesporial cell cuts off the primary parietal cell and forms the parietal layer. The embryo sac develops from a chalazal functional megaspore according to Polygonum type. Endosperm formation is Nuclear, and later becomes cellular. Embryogeny conforms Onagrad type^30^.

### Clearing technique of reproductive cells and structure

Observations of embryological structures and processes were carried out on 23 flowers of the hybrid from which 227 pistils/ovules were studied. Pistils/ovules were at different developmental stages from ovule primordium to ovule with mature gametophyte in flowers after anthesis, namely: megasporocyte, dyad, tetrad, functional megaspore, 2–4 nucleate embryo sac, mature embryo sac stage.

Flowers of *R. circinatus* × *R. fluitans* were not observed to have properly developed zygotic embryos and seeds. Also, no growing pollen tubes were observed in any of the pistils stained with aniline blue. Likewise, pollen grains were not observed on stigmas of clearing pistils. Only in one pistil of the flower from SZL, pollen grains were found growing on the stigma in methyl salicylate cleared material.

Degenerations and abnormalities of the female generative structures and cells occurred in 81% of the examined pistils. The most frequent observations were, as follows: (1) lack of generative cell lineages in properly developed ovules (observed from the youngest ovules to ovules in open flowers); (2) degenerations of whole ovules in properly developed pistils/ovaries; (3) lack of ovule in the ovary. In the male lineage, disturbances and degenerations confirmed earlier observations of pollen grains stained with acetocarmine or Alexander dye.

**Figure 6:**
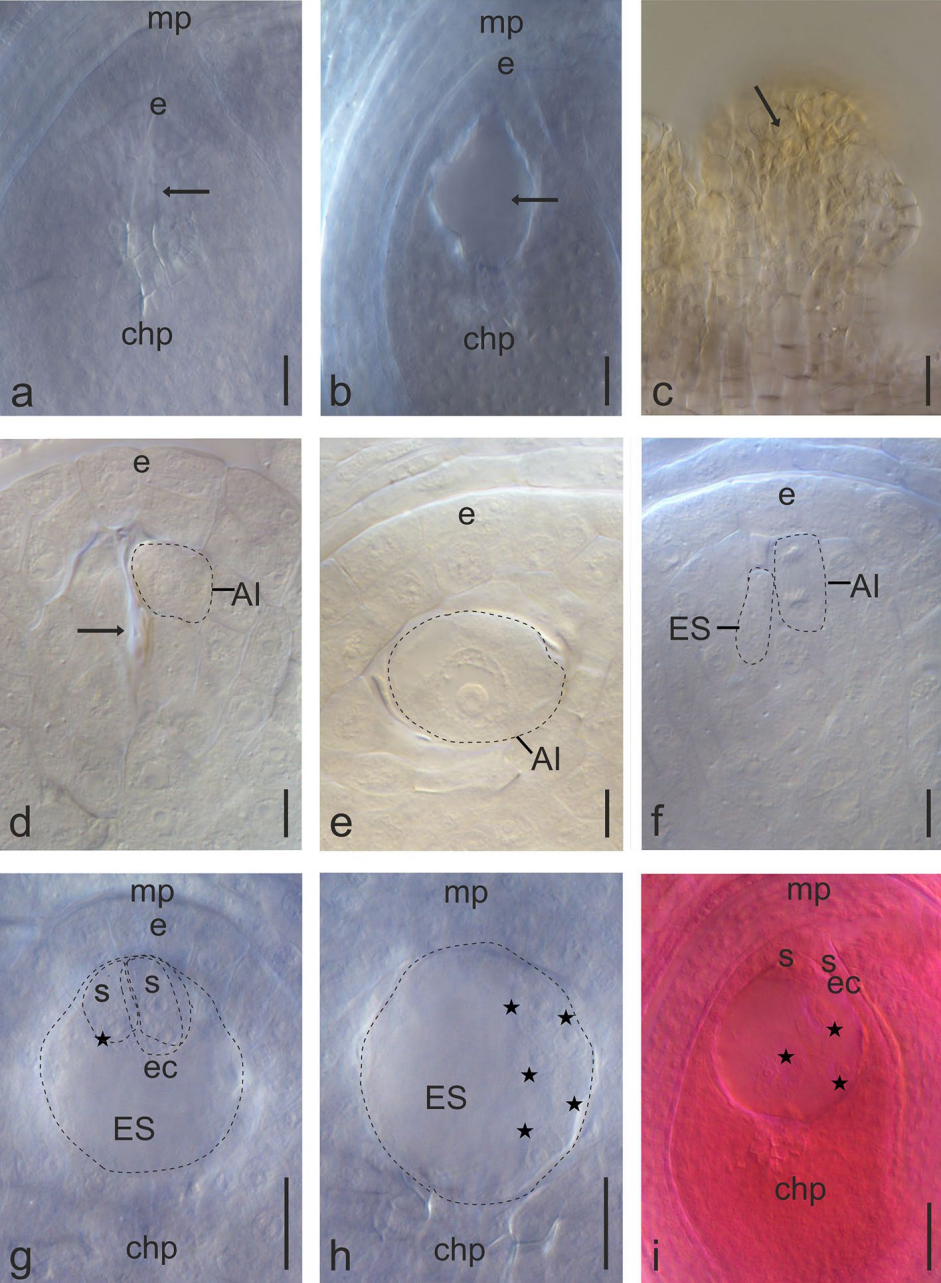
Stages of development of the female generative lineage in the *Ranunculus* hybrid, after cleared with methyl salicylate. Proper developed ovules from flowers at *anthesis* stage, site KON: (**a**) lack of the female gametophyte (arrow); (**b**) empty embryo sac (female gametophyte), (arrow). Site PIL: (**c**) flower at *preanthesis* stage, nucellus degeneration in a young ovule (arrow). Site RUR, young ovules from flowers at *preanthesis* stage: (**d**) degeneration of generative lineage cells (arrow), above, the aposporic initial (AI) is differentiated from the nucellus tissue; (**e**) lack of the meiotic cells lineage. The aposporic initial (AI) is differentiated from the nucellus tissue; (**f**) meiotic and apomeiotic lineage developing together within an ovule, one nucleate meiotic embryo sac (ES), above the telophase in the aposporic initial (AI). Site KON, one ovule from the flower at *anthesis* stage, two successive depths of the ovule, autonomous development of the endosperm in the embryo sac (ES): (**g**) two synergids (s), egg cell (ec), one endosperm nucleus (asterisk); (**h**) four endosperm nuclei (asterisks); (**i**) autonomous development of the endosperm in an ovule from the flower at *postanthesis* stage; in the micropylar pole of the embryo sac (ES) two synergids (s) and the egg cell (ec); free endosperm nuclei (asterisks). *e* epidermis (nucellar cap), *mp* micropylar pole, *chp* chalazal pole. Scar bars (**a**–**c**,**g**,**h**)—25 µm; (**d**–**f**) 10 µm; (**i**) 50 µm.

Completely degenerated anthers, as well as a partial lack of pollen, empty anthers with properly developed stamen walls, and empty, shrunken pollen grains variable in size, were observed (Figs. 5c–h, 6a,b; Supplementary Figs. S4d–I, S5a).

In KAM, PIL, SLU, and ZEL no open flowers were observed. In flower buds, which were inhibited in their development, both male and female structures were in the primordium stage or during degeneration in the early stages of development. In all these cases there was no chance for further development of generative structures and gametophytes (Fig. 6c; Supplementary Fig. S5b).

Ovules considered to be developing properly, not degenerated, were found in 19% of the studied material. In flowers at the *preathesis* stage next to single developing generative lineage, aposporic initials started its development from the nucellus cells. Such cells were larger than surrounding ones, more vacuolated, and differentiated either under the epidermis, next to the generative lineage or below, which were found at the 1-nucleate stage. Mitosis of the aposporic initial was observed in one ovule. Often the initials were accompanied by the degeneration of the generative lineage, and only rarely developed side by side. In the *anthesis* stage, mature embryo sacs were observed, with typically developed seven cells, consisting of the egg apparatus on the micropylar pole (an egg cell, two synergids), the central cell in the middle, and three antipodals on the chalazal pole. Gametophytes with different numbers of cells were also developed. In few gametophytes, a central cell was found with several free nuclei. In others, embryo sacs groups of nuclei at the micropylar pole, or laterally located, were observed. Summarizing the apomictic initials were found in 7% of analyzed ovules. In the next few ovules, double, mature embryo sacs were present, not located in the micropylar-chalazal axis, but transversely to each other, which made interpretation very difficult (Fig. 6d–i; Supplementary Fig. S5c–i).

## Discussion

Hybridization plays an important role in *Batrachium* evolution and spontaneous crosses may occur between species at different ploidy levels. This process results in complex patterns of genetic variation and the formation of new genotypes, which can arise if populations become isolated from a gene flow^16,26,29^. Our results provide a deeper insight into the natural interspecific hybrid *R. circinatus* × *R. fluitans* and its potential role in the evolution of *Batrachium*.

### Genetic variation, propagation, and occurrence of *R. circinatus* × *R. fluitans*

Genetic variation within each of the studied populations of *Ranunculus circinatus* × *R. fluitans* is extremely low with no sign of gene flow between them. A low level of variation within populations is associated with a high level of inter-population variability, resulting in a strong genetic structure correlated with geographic distribution. This finding indicates the recurrent formation of the studied hybrid in at least seven independent hybridization events in different catchment areas. The biological and ecological factors facilitating the formation of the *R. circinatus* × *R. fluitans* hybrid have been discussed more extensively in the paper by Bobrov et al.^24^.

The high genetic similarity of populations from different river catchments (ZEL, the Słupia catchment) and LUP (the Łupawa catchment) shown in this study is likely to be a result of *Batrachium* clumps’ transfer to another river under active conservation actions, which have been carried out over the last 10 years in this area. An alternative explanation for the unusual distribution of genotypes could be the transfer of plant material on kayaks or other floating equipment since recreational boating is well recognized as one of the main factors of plant and animal distribution in freshwaters^31^.

A strong spatial genetic structure is typical for macrophytes, being attributable to the island-like distribution of aquatic habitats, the founder effect, frequent vegetative propagation, dominant self-fertilization, and limited seedling recruitment^32,33^. *Ranunculus circinatus* × *R. fluitans* has sterile seeds, therefore its effective propagation is achieved only vegetatively, mainly by stem fragments within one catchment area. Recently, thanks to molecular evidence, the hybrid has been confirmed in many localities in Central and East Europe^16,23,24^. Due to the frequent occurrence and persistence over time and space, the hybrid is an ecologically important element of river vegetation.

### *R. circinatus* × *R. fluitans* as a triploid bridge

Both parental species are diploid, 2n = 16, even though *R. fluitans* has also been reported as triploid or tetraploid^29,34–37^. The predominately diploid ploidy level of the parental species suggests that the hybrid should also be diploid. However, the chromosome counts of the samples from the river Rurzyca showed that the hybrid *R. circinatus* × *R. fluitans* was triploid, 2n = 24. Koutecký et al.^16^, using molecular identification, reported the same ploidy level from Bavaria, from the novel locality than previously known^25^. The size of the hybrid genome fits well with the sum of *R. fluitans* (two copies) and *R. circinatus* (one copy). The maternal parent of all molecularly investigated hybrid individuals of *R*. *circinatus* × *R*. *fluitans* from Poland, Germany, and Lithuania is invariably *R. fluitans*^16,23,24^. Therefore, in the light of data based on genome size, the studied populations comprising triploid individuals of *R*. *circinatus* × *R*. *fluitans* were formed by an unreduced (diploid), female gamete of *R. fluitans* fertilized by a reduced (haploid) pollen of *R. circinatus.*

Triploids are usually sterile due to genetic incompatibilities compounded by abnormalities during meiosis^22^. The studied hybrid taxon is sterile and likely to be an evolutionary dead-end, even if it is successful locally and ecologically. However, particularly when the chromosome number doubles, sterile hybrids may give rise to new species with selective advantages over their parents and colonize new areas^38^. Here, we observed the partial restoration of fertility of the initially sterile hybrid. The population SZL (Szlamica River) differs from all other populations of *R. circinatus* × *R. fluitans* by its unique genome size, which also shows a different AFLP pattern, sharing the majority of genes with *R. fluitans* (Fig. 2b). The genome size corresponds to a combination of three chromosome sets of *R. fluitans* and one of *R. circinatus* (Table 2), which could explain the gene composition bias towards *R. fluitans*. Morphological characteristics of individuals from SZL are also alike *R. fluitans*, except for the characteristic of the receptacle, which is puberulent (in contrary to glabrous in *R. fluitans*). The percentage of viable pollen grains is 17.18% ± 3.17, which is significantly higher than that observed in the other hybrid populations. Most likely this taxon is an allopolyploid, with two possible modes of origin: (i) by backcrossing triploid *R. circinatus* × *R. fluitans* (2x or 3x gamete) with diploid *R. fluitans* (reduced or unreduced gamete), (ii) through the hybridization of triploid *R. fluitans* (unreduced 3x  gamete) with *R. circinatus* (reduced gamete). We assume the former to be more probable, given the abundance of the triploid *R. circinatus* × *R. fluitans* in the area under study. The tetraploid taxon with the identical presumed mode of origin was recently reported from the Czech Republic as “*R. penicillatus* B”^16^, albeit with slightly different genome size. Possibly these two allotetraploids could have arisen independently from the same parental combination, but the hybridization was followed by different chromosomal rearrangements. This example shows that despite the significant sterility of triploid hybrids, occasionally, further cross-breeding interactions can occur. In the literature, there are many examples, also of the *Ranunculus* genus, where triploid hybrids act as an ‘evolutionary bridge’ between diploids and descendant allopolyploids^39,40^. However, the next steps toward the formation of new *Batrachium* allopolyploids are still insufficiently understood. In this study, aposporic initials were found in 7% of analyzed ovules of triploid *R. circinatus* × *R. fluitans.* In the other *Batrachium* hybrid, namely *R. fluitans* × *R. peltatus,* autonomous development of endosperm without fertilization was observed^41^. These observations suggest a developmental pathway of partial apomixis, which is a common strategy of polyploid plants to escape sexual-based F1sterility^40^.

### The ability of *R. fluitans *to form polyploids and its role in reticulate evolution of sect. *Batrachium*

In this study, the independent formation of the hybrid *R. circinatus* × *R. fluitans* is striking in the context of the previous viewpoint of Cook^26^, who, based on crossing experiments suggested that diploid *Batrachium* species hybridize only exceptionally. Recently, however, Prančl et al.^29^ and Koutecký et al.^16^ showed that hybridization between diploid species is more common than previously thought and which our study confirmed.

Except for diploid also triploid and tetraploid forms of *R. fluitans* are reported^29,34,36^. In this study, one population of *R. fluitans* turned out to be triploid (KAC). Molecular data with no traces of hybridization together with morphologically consistent characters of cytotypes supported the hypothesis that triploid forms of *R. fluitans* are autopolyploid [^16^, and this study].

In contrary to triploid forms, the formation of tetraploids in *R. fluitans* reported to date is uncertain. This ploidy level was reported from several localities in Europe (e.g. Ref.^26^ from Britain) based on the chromosome counts of morphologically defined individuals. Due to seasonal phenotypic plasticity and frequent hybridization, morphological identification of *Batrachium* taxa can be misleading^13^. In Lithuania, tetraploid ‘*R. fluitans*’ was reported from the rivers in the Vilnius surrounding, i.e. Vokė and Vilnia^36^. In these rivers, based on the molecular survey, the hybrid *R*. *circinatus* × *R*. *fluitans* was confirmed, and at the same time, *R. fluitans* was not found^24^. Both taxa are morphologically quite similar, with the biggest difference in the character of a receptacle, which is glabrous in *R. fluitans* and puberulent in *R*. *circinatus* × *R*. *fluitans*. However, Bobrov et al.^24^, in the morphological description of *R*. *circinatus* × *R*. *fluitans* from Lithuanian rivers reported a variable character of a receptacle, puberulent to almost glabrous. Moreover, both taxa from these localities, tetraploid, putative ‘*R. fluitans*’*,* and *R*. *circinatus* × *R*. *fluitans* were proved to be completely sterile. It is highly probable that tetraploid individuals from Vokė and Vilnia rivers, identified solely on the basis of morphological features as ‘*R. fluitans*’ by Turała-Szybowska^36^ represented in fact the hybrid *R. circinatus* × *R. fluitans*, as indicated in recent research by Bobrov et al.^24^. Recently, a tetraploid cytotype of *R. fluitans* from the Czech Republic has also been questioned^29^.

The triploid forms of *R. fluitans*, as well as the relatively frequent occurrence of the hybrid *R*. *circinatus* × *R*. *fluitans*, which, as documented here, arises by the fertilization of diploid female gametes of *R. fluitans*, gives evidence that *R. fluitans* has the ability to the formation of unreduced megagametophytes. The most taxonomically complicated group of taxa within *Batrachium*, namely *R. penicillatus* complex, is a collection of allopolyploids that have arisen from hybrids between *R. fluitans* from one side, and *R. peltatus, R. trichophyllus, R. aquatilis,* and *R. circinatus* from the other^13,26^. The evolutionary history of *R. penicillatus* group is shaped by different kinds of hybridization events, including multiple backcrosses, episodes of introgression, polyploidization, and whole-genome evolution^13,16,26^. The key role of *R. fluitans* is most likely attributed to its ability to produce unreduced megagametophytes (apomeiosis), which is regarded as one of the main forces of polyploid formation in angiosperms reviewed in Ref.^42^.

### Taxonomic consequences of hybridization, introgression, polyploidization, and extreme plasticity in *Batrachium*

A reticulate pattern of evolution due to hybridization, introgression, and polyploidization, as well as extreme phenotypic plasticity, can be a result of adaptation to the aquatic environment. This can blur the boundaries between taxa in *Ranunculus* section *Batrachium.* Gluchoff-Fiasson et al.^43^ suggested that at least some of the widely distributed *Batrachium* taxa that are given the same, morphologically based, botanical name, actually represent totally different, convergent, entities that have arisen independently from separate lineages. With the advent of advanced molecular methods, increasingly more data support this hypothesis and show that morphological species are in fact polytypic^14–16,29^.

On the other hand, there is still insufficient molecular evidence and modern taxonomic data that can define taxa adequately, especially in the context of the whole taxa ranges and concerning areas of great evolutionary importance for the section, e.g., the Mediterranean.

Diploid populations of *R. fluitans* forming intermediate leaves (“moose antlers-like”) have been reported in the past from Germany as "*R. fluitans* hybrid 1"^16^. We confirmed that such populations fall within the variability of *R. fluitans*, since its individuals show no admixture of any other species in the genetic analyses. This finding contradicts the traditional concept of *R. fluitans* as a strictly homophyllous species^13^.

Naturally formed hybrids such as *R*. *circinatus* × *R*. *fluitans* can be evolutionarily important by producing fertile offspring. Due to the high morphological and genetic variation including differences in ploidy levels (the case of SZL population) the taxonomic treatment of such complex lineages is difficult. No general agreement exists beyond the prescriptions in Chapter H of the Shenzhen Code^44^. Before assigning a formal binomen to *Batrachium* hybrids, genetic and karyological studies are imperative to identify both the parental species, ploidy level, karyotype, and the DNA content.

## Conclusions

Overall, the results demonstrate a strong genetic structure of *R. circinatus* × *R. fluitans* in Poland, which is attributed to independent hybridization events, sterility of hybrid individuals, vegetative propagation, and isolation through the geographical distance within populations.

The hybrid *R. circinatus* × *R. fluitans* is a sterile triploid; however, as documented in this study, it may participate in subsequent hybridization events, resulting in a ploidy change that can lead to spontaneous fertility recovery.

The ability to produce unreduced female gametes by the hybrid *R. circinatus* × *R. fluitans* and the parental species *R. fluitans*, probably has the same origin (apospory) and is an important evolutionary mechanism in the *Batrachium* group that could give rise to novel taxa.

## Materials and methods

### Plant material

In total, 36 riverine natural populations of *Batrachium* occurring in Poland, Central Europe, were studied, 14 of a hybrid *Ranunculus circinatus* × *R. fluitans,* 11 of *R. circinatus,* and 12 of *R. fluitans* (Fig. 1a). A detailed list of the study locations is provided as Supplementary Table S1. (1-)5(-6) individuals per population were used for AFLP analysis, which represents 165 individuals in total. Reference herbarium specimens for individuals sampled for AFLP analysis are deposited in the Herbarium of the Institute of Botany, Jagiellonian University, Kraków (KRA). The authors confirmed that the collection of plant samples has been conducted in accordance with local legislation, under the Field-Work Permits: WPN-I.6400.25.2011.KA, WPN.6402.2.26.2011.DJB, WPN.6402.45.2011.MG and WOPN-OOP.6402.2.17.2011.WP.

### DNA extraction and Amplified Fragment Length Polymorphism (AFLP) fingerprinting analysis

Samples were collected in a field from separate *Batrachium* individuals at a distance of at least two meters from each other. Each sample consisted of fresh leaves, which were stored in plastic tubes with silica gel and preserved at room temperature till DNA isolation. Total DNA was extracted from ca. 10 mg of dried plant material using Mixer Mill 300 (Retsch) and the DNeasy Plant Mini Kit (Qiagen) according to the manufacturer’s protocol (final elution step was carried out using 2 × 50 μL elution buffer). DNA quality and concentration were estimated against λ-DNA on 1% agarose gel stained with ethidium bromide.

AFLP fingerprinting analysis followed the procedure of Vos et al.^45^ with modifications as described in detail by Ronikier et al.^46^. Double-digestion of DNA was performed using *Eco*RI and *Mse*I enzymes. Subsequently, double-stranded *Eco*RI/*Mse*I adapters were ligated to the digested DNA using T4 DNA ligase. Polymerase chain reaction (PCR) amplifications were performed on a GeneAmp 9700 thermal cycler (Applied Biosystems). Preselective PCR used *Eco*RI-A and *Mse*I-C primers. Subsequently, selective PCR reactions were performed using 5'-fluorescence-labelled *Eco*RI selective primers (6-FAM). Selective amplification products were separated using 36-cm capillaries and POP 4 polymer (Applied Biosystems) with Genescan ROX-500 (Applied Biosystems) internal size standard on an ABI PRISM 3100-*Avant* automated sequencer (Applied Biosystems). At the preliminary screening step, 12 selective primer pair combinations were tested and evaluated for clarity of profiles (i.e. prevalence of well-separated markers), and number and repeatability of polymorphic markers. Three primer pairs were selected for the final analysis: *Eco-*ACC/*Mse-*CAG, *Eco-*ACA/*Mse-*CAT and *Eco-*AGA/ *Mse-*CTG.

AFLP fragments were manually scored using Genographer 2.1 (http://sourceforge.net/projects/genographer). The reproducibility of AFLP markers was assessed using 12 within-plate and four between-plate samples replicates carried in parallel through all reaction steps. The error rate was calculated as the percentage of mismatches in the scoring of AFLP profiles of replicated individuals. Only markers that scored unambiguously (i.e. well separated) and repeatedly in the duplicates were considered. AFLP fragments in the size range of 40–500 bp were scored and assembled in a binary presence/absence matrix.

### Molecular variation and genetic structure

The overall genetic relationships among the study populations of *R. circinatus* × *R. fluitans* and its parental species *R. circinatus* and *R. fluitans* were explored using principal coordinate analysis (PCoA) based on Nei's genetic distances^47^.

The analysis of molecular variance (AMOVA) was based on groups defined a priori (populations) separated by localities (rivers). AMOVA was based on the pairwise squared Euclidean distance among molecular phenotypes. Significance levels were determined by 1000 permutations. AMOVA and PhiPT index were calculated using GenAlEx 6.5 software.

The most likely number of population clusters was estimated in the STRUCTURE 2.3.2 software^28^, with the model for dominant markers^48^, correlated allele frequencies, and admixture. STRUCTURE uses a Bayesian clustering algorithm to assign individuals to a specified number of clusters (*K* value). Analysis was run with 200,000 burn-in cycles and 1,000,000 sampling MCMC cycles, for *K* value between 2 and 15, in five replicates each. CLUMPAK^49^ was used to determine the optimal *K* value using the Δ*K* method of Evanno et al.^27^, and to align all optimum *K* STRUCTURE runs to the permutation with the highest H-value and visualize the output.

For reconstructing the relationships among the populations and identifying population clusters, the NeighbourNet analysis was used with uncorrected p-distances, as implemented in Splits-Tree4 version 4.11.3^50^. Bootstrap support for splits was calculated using 1,000 replicates.

### Mantel test

The Mantel test^51^ with 9999 permutations was performed to determine the relationship and statistical correlation between the two matrices of distance, i.e. the genetic distance matrix (unbiased Nei's genetic distances) and the geographical distance matrix. This was done in order to determine whether between-populations similarities in terms of genetic distance and geographical distance were significantly interrelated. The analysis was done for the whole data matrix and for *R. circinatus* × *R. fluitans*, separately. The analyses were made using GenAlEx 6.5 software^52^.

### Chromosome number determination

As hybrid individuals did not produce seeds, an attempt was made to count the chromosomes from the roots of adult specimens. The material for karyological investigations was fixed in situ and ex situ from the plants cultivated in the experimental garden. A different sample processing protocol was used for each type of material.

The plant individual from RUR population was cultivated in a tank in the experimental garden of the Institute of Botany, Průhonice, Czech Republic. In September and October 2018 roots from cultivated material were prepared according to the procedure successfully applied in previous karyological studies of *Batrachium* and described in Ref.^29^. The preparations were examined under an Olympus BX 51 microscope equipped with DP-71 Olympus digital camera with the cellSense imaging software [Ver. 2.3] (Olympus Corp.).To get more material for karyology other roots were collected in situ in July 2022 (populations KAM, PIL, SLU, GWD, PLY, RUR, ZEL, KON) and prepared as described in Ref.^53^, namely: incubated in a saturated aqueous solution of 8-hydroxyquinoline for 4 h, fixed in a mixture of ethanol 96% and glacial acetic acid (3:1, v/v) and stored at 4 °C until further processing. Next, the fixed roots were stained in 2% acetic orcein (Fluka, Switzerland) for 4–6 days at room temperature. The stained roots were transferred to 45% acetic acid and heated to boiling over a flame, thereafter root tip meristems were cut off and squashed in a drop of 45% acetic acid. The coverslip was removed after freezing in liquid nitrogen and the slide was thoroughly air-dried and mounted in Entellan. The late prophase and metaphase chromosomes were counted and photographed using Nikon Eclipse E400 light microscope equipped with a camera and NIS-Elements Viewer imaging software ver. 4.00 (Tokyo, Japan).

### Genome size

Genome size was estimated for 118 individuals using flow cytometry. The identical procedure and laboratory equipment was used as in Prančl et al.^29^ and Koutecký et al.^16^. The genome size of each individual was estimated using propidium iodide as a stain and using *Bellis perennis* L. (2C = 3.38 pg; ^54^) as the internal standard. Selected individuals (usually 3 for each population; in total 87 samples) were analyzed (2–)3 times on different days; if the range of variation of the repeated measurements exceeded a 2% threshold, the outlying value was discarded, and the sample re-analyzed. Only these repeatedly measured individuals were used for the calculation of the genome size statistics of particular taxa (Table 2).

### Embryological techniques

In total, 53 flowers in different developmental stages (*preanthesis, anthesis, postanthesis*) were collected in July 2022, fixed in situ in a mixture of ethanol 96% and glacial acetic acid (3:1, v/v), and stored at 4 °C until used. At the collecting time, flowers at the *anthesis* stage were present only at two sites, KON (21) and RUR (4). Stages representing *preathesis* and *postanthesis* were also present (KON 3 and 2, respectively; RUR 16 and 7, respectively). In the remaining sites, several undeveloped flower buds were found, in KAM (6), PIL (17), SLU (11), ZEL (3).

### Viability of pollen

Pollen viability (stainability) was assessed with Alexander dye^55^ and acetocarmine^56^. Eighteen flowers collected at KON and RUR were tested. Fixed pollen grains were removed from the anthers directly onto the glass slide to a drop of reagent. Viable pollen stained red–purple, non-viable green using Alexander dye. For acetocarmine, viable pollen stained red, and non-viable remained colourless. Additionally, for observation of earlier developmental stages (microsporogenesis) three flower buds were tested from PIL, RUR, and SLU. The pollen viability was performed on herbarium material from the SZL site. Twelve flowers were soaked in 70% ethanol for 5 days. Then the same procedure was followed as for pollen fixed in the field. Pollen grains were observed, and the diameter of stained pollen grains was measured using Nikon Eclipse E400 light microscope equipped with camera and NIS-Elements Viewer imaging software ver. 4.00 (Tokyo, Japan).

### Clearing of flowers

For embryological processes observation, 23 flowers (KAM, KON, PIL, RUR) were cleared using methyl salicylate method, and divided into two groups. I. Flowers at *preanthesis* and *anthesis* stages were cleared in a mixture of 100% ethanol and methyl salicylate (Sigma-Aldrich) in proportions 3:1; 1:1; 1:3, 0:3 two hours each change. II. At *postanthesis* stages prior to clearing, pre-softening of the tissues using Schiff reagent was applied. Cleared pistils and/or anthers were transferred onto Ray chambers in a drop of methyl salicylate and observed under Nikon Eclipse Ni light microscope with Nomarski differential interference contrast (DIC) optics, equipped with camera Nikon DS-Filc and NIS-Elements BR Viewer imaging software ver. 4.10 (^57^, slightly modified). For the study, 10 randomly selected ovules from each flower were analyzed.

### Pollen tube growth

For pollen tube growth observations twenty randomly selected pistils from open flowers, were stained in 0.1% aniline blue as described in Kwiatkowska et al.^58^. Then pistils were slightly incised under a stereoscope microscope, ovaries were squashed on slides in the phosphate buffer and observed under Nikon Eclipse E400 microscope with fluorescence excitation at 330–380 nm.

## Supplementary Information

Supplementary Information.

## Data Availability

The datasets used and/or analyzed during the current study are available from the corresponding authors upon request. Herbarium specimens of *R. circinatus* × *R*. *fluitans* are preserved in KRA.
