# Supplementary material for: Origin, genetic structure and evolutionary potential of the natural hybrid Ranunculus circinatus × R. fluitans
Source: Sci Rep. 2023 Jun 3;13:9030. doi: 10.1038/s41598-023-36253-7 (PMC10239522; doi:10.1038/s41598-023-36253-7)
Supplement: Supplementary file 1 — Supplementary Information. [file 41598_2023_36253_MOESM1_ESM.pdf]

Supplementary data

Origin, genetic structure and evolutionary potential of the natural hybrid *Ranunculus circinatus* × *R. fluitans*

J. Zalewska-Gałosz[1]*, M. Kwiatkowska[1], J. Prančl[2], K. Skubała[1], M. Lučanová[2, 4], D. Gebler[3], K. Szoszkiewicz[3]

[1]Institute of Botany, Faculty of Biology, Jagiellonian University, Gronostajowa 3, 30-387, Kraków, Poland

[2]Czech Academy of Sciences, Insitute of Botany, Zámek 1, 252 43 Průhonice, Czech Republic

[3]Department of Ecology and Environmental Protection, Poznan University of Life Sciences, Wojska Polskiego 28, 60-637, Poznań, Poland

[4]Department of Botany, Faculty of Science, University of South Bohemia, Branišovská 31, CZ-370 05 České Budějovice, Czech Republic

*joanna.zalewska-galosz@uj.edu.pl

Supplementary Table S1. A list of populations studied including their taxonomic affiliations, location with geographical coordinates (WGS84), population codes and numbers, as well as numbers of DNA samples

**Taxon**	**Locality**	**Latitude (N)**	**Longitude (E)**	**Population code**	**Population number**	**No of DNA samples**	**No of DNA samples in Figures**
R. fluitans	Bóbr River, Trzcińsko	50 52 42.1	15 51 05.5	TRZ	1	A1-A6	1-6
R. fluitans	Lenka River, Słońsk	52 34 14.5	14 48 19.9	LEN	12	A88-A92	47-51
R. fluitans	Kaczawa River, Złotoryja	51 07 42.3	15 55 00.9	KAC	3	A331-A333	12-14
R. fluitans	Nysa Kłodzka River, Bardo	50 30 16.09	16 44 12.19	NYS	4	A335-A339	15-19
R. fluitans	Osobłoga River, Głogówek	50 21 09.8	17 50 58.8	OSO	5	A341-A345	20-24
R. fluitans	Wieprza River, Korzybie	54 18 20.2	16 52 00.8	WIE	6	A59-A63	25-29
R. fluitans	Kanał Miejski Canal, Sławno	54 21 43.0	16 40 57.0	SLA	7	A64-A68	30-34
R. fluitans	Nysa Kłodzka River, Krosnowice	50 23 48	16 37 36.0	KRO	8	A7	35
R. fluitans	Bystrzyca Dusznicka River, Kłodzko	50 25 00	16 38 29.9	BYS	9	A8	36
R. fluitans	Drawa River near Mostniki	53 01 11.3	15 56 34.5	DRA	10	A74-A78	37-41
R. fluitans	Postomia River, Sulęcin	52 26 26.0	15 06 37.7	POS	11	A83-A87	42-46
R. circinatus × *R. fluitans*	Rygol, Szlamica River	53 53 46.67	23 26 53.10	SZL	2	A281-A285	7-11
R. circinatus × *R. fluitans*	Kończak River, Stobnica	52 42 15.3	16 36 17.7	KON	13	A10-A14	52-56
R. circinatus × *R. fluitans*	Rurzyca River, Krępsko	53 15 49.9	16 46 33.3	RUR	14	A17-A21	57-61
R. circinatus × *R. fluitans*	Płytnica River, Płytnica	53 26 58.1	19 40 15.6	PLY	15	A25-29	63-67
R. circinatus × *R. fluitans*	Płytnica River, Płytnica 1	53 18 25.6	16 47 29.9	PLT	16	A108-A112	68-72
R. circinatus × *R. fluitans*	Piława River, Szwecja	53 21 06.6	16 34 11.7	PIL	17	A35-A39	73-77
R. circinatus × *R. fluitans*	Kamienna River, Skarszów Dolny	54 21 26.6	17 06 45.5	KAM	18	A44-48	78-82
R. circinatus × *R. fluitans*	Słupia River, Lubuń	54 21 37.91	17 05 8.11	SLU	19	A103-A107	84-88
R. circinatus × *R. fluitans*	Gwda River, Piła	53 08 47.3	16 45 07.45	GWD	20	A16; A93-A97	89-94
R. circinatus × *R. fluitans*	Żelkowa Woda River, Żelkowo	54 21 22.8	17 06 04.2	ZEL	21	A49-A54	83; 95-98
R. circinatus × *R. fluitans*	Łupawa River, Smołdzino	54 39 43.9	17 12 48.7	LUP	22	A54-A58	99-103

R. circinatus × *R. fluitans*	Drwęca River, Golub-Dobrzyń	53 06 45.3	19 03 06.9	DRW	23	A98-A102	104-108
R. circinatus × *R. fluitans*	Drawa River, Zatom	53 08 27.2	15 50 54.8	ZAT	24	A69-A73	109-114
R. circinatus × *R. fluitans*	Drawa River, Barnimie	53 08 52.2	15 52 13.5	BAR	25	A79-A82	115-118
R. circinatus	Rurzyca River, Krępsko	53 15 49.9	16 46 33.3	RUR	26	A23-A24	119-120
R. circinatus	Czarna River, Sochonie	53 13 01.7	23 10 49.5	SOC	27	A277-A280	121-124
R. circinatus	Rospuda River, Filipów Pierwszy	54 10 32.3	22 37 38.65	ROS	28	A288-A292	125-129
R. circinatus	Marózka River, Orzechowo	53 33 20.6	20 26 10.7	MAR	29	A301-A305	130-135
R. circinatus	unnamed rivulet, Mroczno	53 20 56.6	19 44 16.1	MRO	30	A306-A310	136-140
R. circinatus	Obrzański Kanał Południowy Canal, Rudno	52 0 25.9	15 59 17.57	OBR	31	A312-A316	141-145
R. circinatus	Mierzawa River, Pawłowice	50 30 15.15	20 27 44.3	MIE	32	A319-A323	146-150
R. circinatus	Piława River, Szwecja	53 21 6.6	16 34 11.7	PIL	33	A30-A34	151-155
R. circinatus	Nida River, Żerniki	50 45 16.81	20 24 21.6	NID	34	A324-A328	156-160
R. circinatus	Kłonecznica River, Budy	53 58 45.6	17 30 44.7	KLO	35	A40-A43	161-164
R. circinatus	Gidle, Kanał Lodowy	50 57 18.1	19 28 12.4	GID	36	A09	165

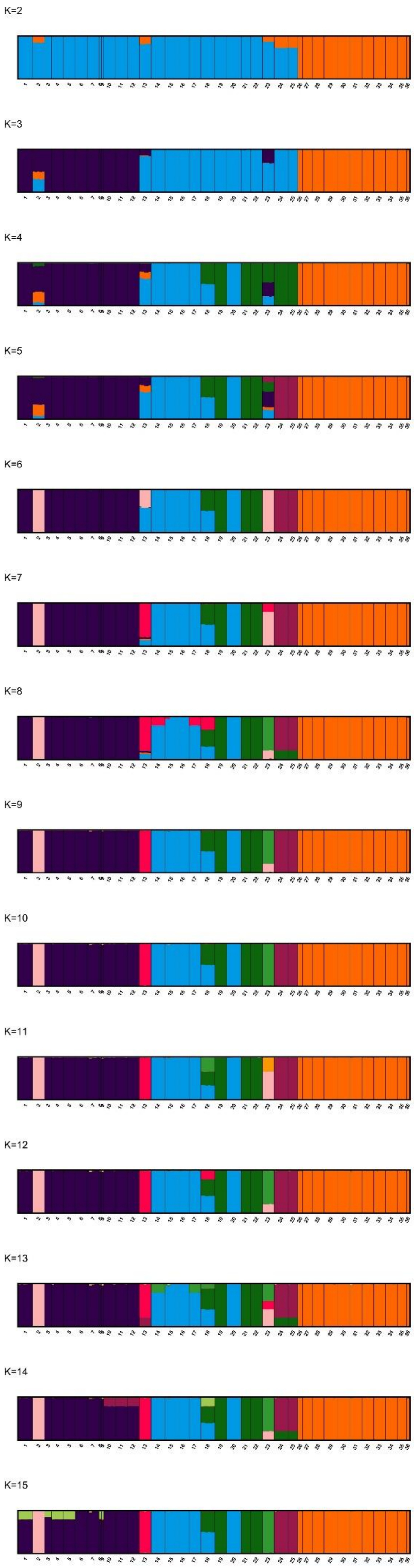
K=2
K=3
K=4
K=5
K=6
K=7
K=8
K=9
K=10
K=11
K=12
K=13
K=14
K=15

Supplementary Figure S1. Major models for *K* 2–15 visualized in CLUMPAK. Populations numbers are explained in Supplementary Table S1

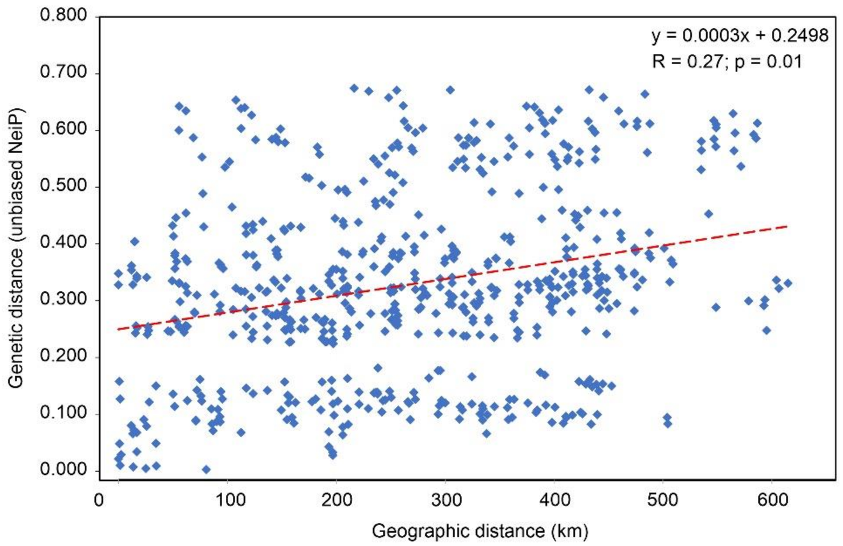

Supplementary Figure S2. Scatterplot showing the results of the Mantel test of the correlation between the similarity matrix of genetic distances (unbiased Nei's genetic distances) and that of geographic distances (km). Based on the whole data set.

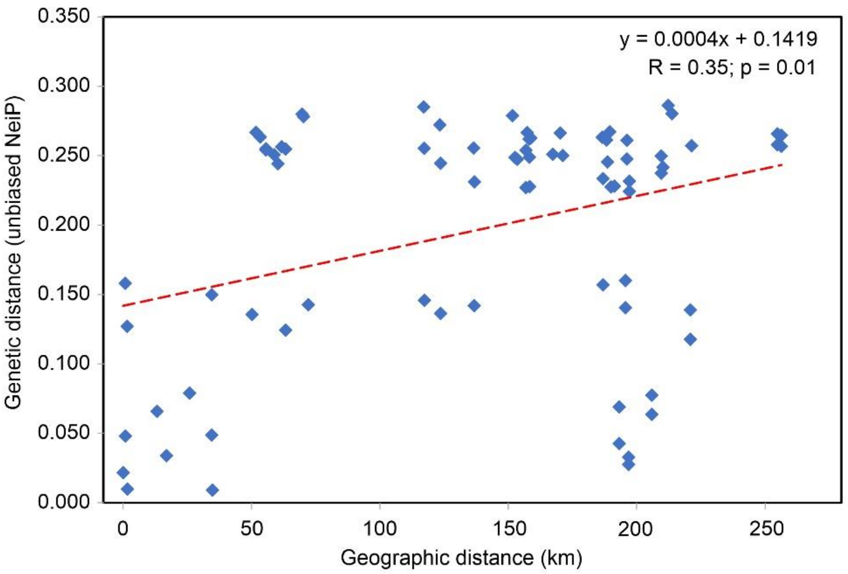

Supplementary Figure S3. Scatterplot showing the results of the Mantel test of the correlation between the similarity matrix of genetic distances of *R. circinatus* × *R. fluitans* populations (unbiased Nei's genetic distances) and that of geographic distances (km).

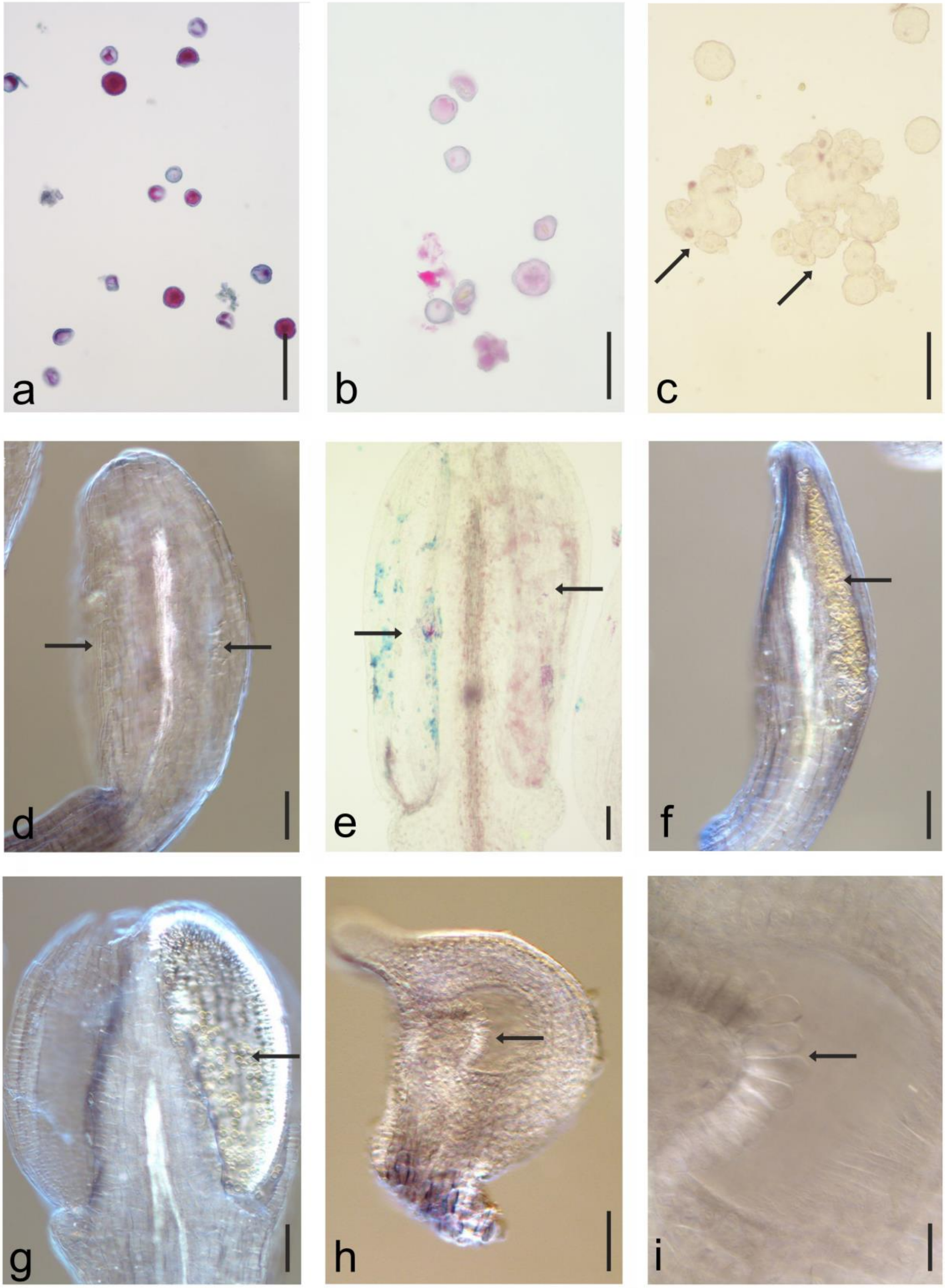

Supplementary Figure S4. Pollen grains stainability and generative structures development in *Ranunculus* hybrid. **a-b** – stainability/viability of pollen grains after staining with Alexander dye; red stained viable pollen grains, green stained non-viable pollen grains; **a** - site SZL, **b** –

site KON; **c** – groups of agglomerated, unstained pollen (arrows) after staining with acetocarmine, site KON. Anther structure after cleared with: methyl salicylate (**d**) and Alexander dye (**f-e**): **d** – anther from the flower at *preanthesis* stage; empty pollen sacs (arrows); site PIL. **e** – anther from the flower at *preanthesis* stage; degenerated generative cells lineage in the pollen sacs (arrows); **f-g** – anther from the flower at *anthesis* stage; site KON. **f** – compressed anthers with pollen grains in one pollen sac (arrow); Improperly developed anther wall; **g** – empty pollen sac, the second filled with pollen grains (arrow); properly developed walls with a fibrous endothelium; **h-i** – pistil structure after cleared with methyl salicylate, from the flower at *preanthesis* stage, site PIL; **h** – pistil without an ovule (arrow); **i** – the same ovule enlarged. Instead of an ovule, finger-like elongated cells grew on the placenta (arrow). Scale bars: **a** – 100µm; **b**, **c** – 50µm; **d-h** – 100µm; **i** – 25 µm.

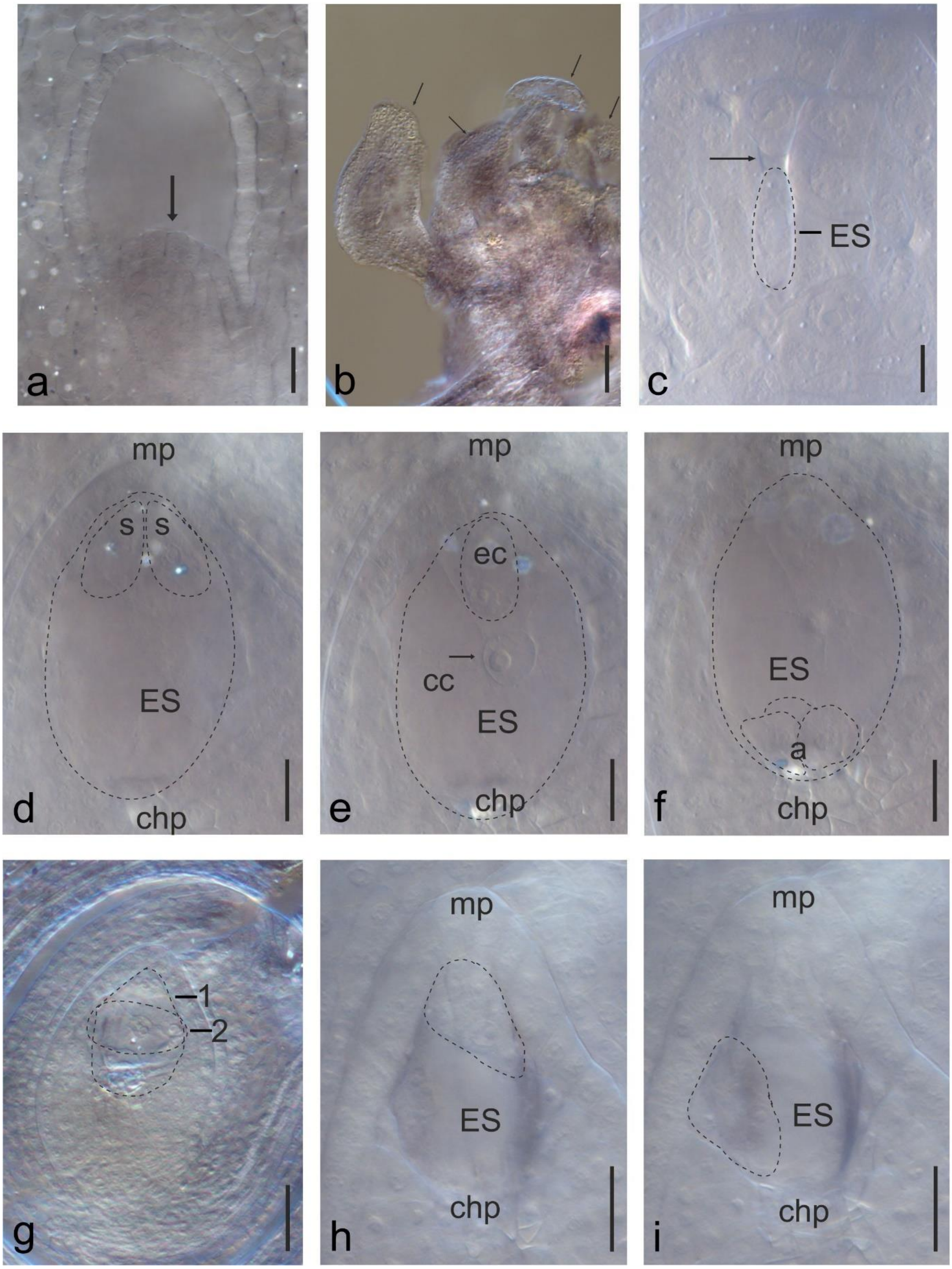

Supplementary Figure S5. Stages of development of the female generative lineage in the *Ranunculus* hybrid after cleared with methyl salicylate. **a-c** – ovules from flowers at *preanthesis* stage; **a** – lack of an ovule (arrow) growing from placenta in the ovary, site RUR; **b** – young

pistils in a stage of the development inhibition (arrows), site PIL; **c** – one nucleate meiotic embryo sac (ES), above, a trace after degenerated megaspores (arrow), Site RUR; **d-i** – ovules from flowers at *anthesis* stage; **d-f** – three successive depths of the mature embryo sac (ES), site KON; two synergid (s), egg cell (eg), cc – central cell with the secondary nucleus (arrow), a – three antipodals; g – two mature embryo sacs in the ovule. (1) – one in the micropylar-chalazal axis, and (2) second embryo sac arranged across, site KON; **h-i** – two successive depths of the mature embryo sac (ES), site KON; autonomous endosperm development; two groups of free nuclei - one at the micropylar pole, the other located laterally; mp – micropylar pole and chp – chalazal pole of the embryo sac. Scale bars: **a, d, f, h-i** – 25µm; **b** – 10µm; **c** – 100µm; **g** – 50µm.
